# Endoscopic submucosal dissection for a metachronous superficial epiglottic lesion with nasotracheal intubation and floss traction

**DOI:** 10.1055/a-2882-8354

**Published:** 2026-06-16

**Authors:** Chenchen Zhang, Honglei Wu, Daoyu Tao, Zhaosheng Chen

**Affiliations:** 1 Department of Gastrointestinal Endoscopy Center, the Second Qilu Hospital, the Second Clinical Medical School 12589 Shandong UniversityJinanShandongChina; 2 Department of Pathology, the Second Qilu Hospital, the Second Clinical Medical School 12589 Shandong UniversityJinanShandongChina

A 65-year-old man presented with a lesion on the lingual epiglottis during routine surveillance. Six years earlier, he had undergone partial laryngopharyngectomy for hypopharyngeal squamous cell carcinomas (SCCs; T1N1M0). Subsequently, two metachronous superficial esophageal SCCs were resected by endoscopic submucosal dissection (ESD) 3 and 2 years ago. He had a long-standing history of heavy smoking and alcohol use, recently reduced but ongoing.

**Fig. 1:**
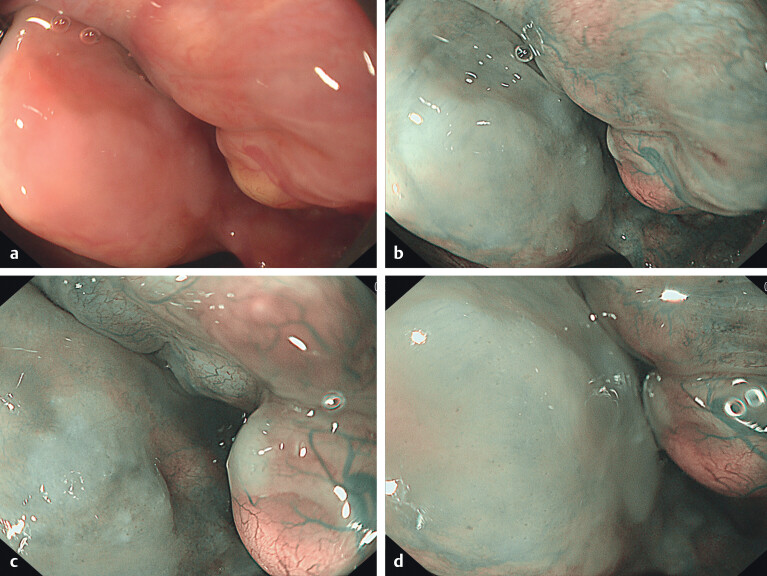
Endoscopic features of the lesion. ( **a** ) A white light view: a well-demarcated whitish lesion, extending from the right vallecula to the free margin; ( **b–d** ) NBI with magnification: whitish plaques with distinct borders, and vessels in the whitish area are poorly visualized. NBI, narrowband imaging.

**Fig. 2:**
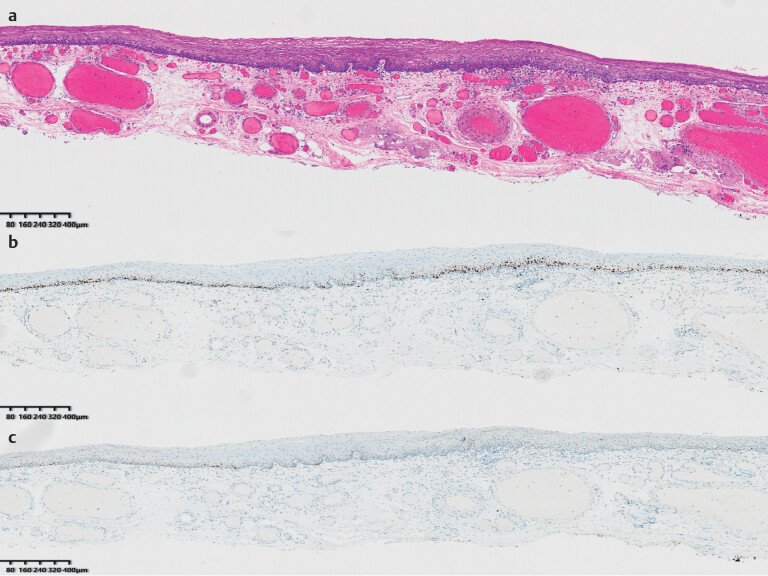
Histopathological findings of the endoscopically dissected specimen. ( **a** ) HE staining (4×); basal layer positivity for Ki67 ( **b** ) and with wild‑type p53 ( **c** ).

Surveillance endoscopy revealed a 1.5 cm, whitish lesion with distinct borders, extending from the right vallecula to the free margin ( Fig 1a ). Narrowband imaging (NBI) with magnification showed a vague vascular pattern with whitish plaques ( Fig. 1b–d ). Biopsy confirmed high grade intraepithelial neoplasia (HGIN), and computed tomography revealed no metastasis. Under general anesthesia with orotracheal intubation, we performed ESD using a water-jet knife. After NBI marking and Lugol staining, the incision was carried circumferentially with the strategy as depicted in the video, but incision of the free margin was tricky with the orotracheal tube. To create more working space, we replaced orotracheal intubation with nasotracheal intubation under nasal endoscopic guidance. After incising the free margin of the epiglottis, floss traction exposed the resection plane, allowing the complete resection of the lesion. The ESD procedure was completed in 45 minutes ( Video 1 ). No intra- or post-procedural complications were observed. Histopathology confirmed HGIN with negative margins ( Fig. 2 ). The patient swallowed normally at 2 months, and no postoperative dilations or other interventions were needed.

**Video 1** Gastroscopy showing endoscopic features of the lesion and the procedure of endoscopic submucosal dissection (ESD).


This case highlights two lessons. First, field cancerization demands rigorous surveillance in high-risk patients with head and neck SCCs, particularly in blind spots such as the epiglottis and pyriform sinuses. 1​ ​2​ Second, the procedure succeeded with key technical adaptations—nasotracheal intubation to optimize working space, floss traction to aid visualization, and a water-jet functional Knife to improve efficiency. ESD is feasible even for challenging epiglottic lesions involving the free margin.

Endoscopy_UCTN_Code_TTT_1AO_2AG_3AD
